# Analyzing innovative policies and practices for palliative care in Portugal: a qualitative study

**DOI:** 10.1186/s12904-024-01556-7

**Published:** 2024-09-11

**Authors:** Marcelle Miranda da Silva, Audrei Castro Telles, Cristina Lavareda Baixinho, Eunice Sá, Andreia Costa, Maria Adriana Pereira Henriques

**Affiliations:** 1 Escola de Enfermagem Anna Nery (EEAN), Universidade Federal do Rio de Janeiro (UFRJ), Afonso Cavalcanti Street, Rio de Janeiro, 21211-110 RJ Brazil; 2 Nursing Research, Innovation and Development Centre of Lisbon (CIDNUR), Prof Egas Moniz Avenue, Lisbon, 1600-190 Portugal; 3 Instituto de Saúde Ambiental (ISAMB), Faculty of Medicine, Universidade de Lisboa, Prof Egas Moniz Avenue, Lisbon, 1649-028 Portugal; 4 Laboratório Associado TERRA, Faculty of Medicine, Universidade de Lisboa, Prof Egas Moniz Avenue, Lisbon, 1649-028 Portugal

**Keywords:** Palliative Care, Hospice Care, Primary Health Care, Health Policy, Delivery of Health Care, Portugal

## Abstract

**Background:**

Countries with formal policies for palliative care, and advanced and integrated practices in this field, such as Portugal, face challenges in achieving excellence in care, particularly in home-based assistance. Issues include care coordination among providers, confusion regarding the roles of each health care professional in the network, and a lack of monitoring and evaluation of actions. Our objective was to analyze the implementation of palliative care in primary health care in Portugal.

**Methods:**

We conducted a qualitative, descriptive, and exploratory study in Portugal involving health care professionals with experience in palliative care. The data were collected through semistructured interviews and focus groups between March and October 2023. Eighteen health care professionals participated. We used the Alceste software for lexicographic analysis. The research was authorized by an Ethics Committee.

**Results:**

Four classes were identified; classes 1 and 2, comprising 77% of the corpus, addressed the study objectives. Participants highlighted inequitable access, strategic development plans with unattainable short-term goals; and low literacy. They emphasized the importance of legislation, professional training initiatives for generalist palliative care at home, and early referral. Home-based challenges included professionals’ lack of exclusive dedication, absence of 24/7 coverage, and unavailability of capable family caregivers. The networks’ response to hospital admissions and patient transitions from hospital to home, with access to the specialized team, was also inadequate.

**Conclusions:**

Health care professionals aim to increase patients’ time spent at home, reduce emergency department visits, and minimize hospitalizations by leveraging the resources of the national palliative care network. In addition to investments to sustain network implementation and legally guaranteed palliative care rights, the country must focus on measurable indicators for evaluating and monitoring actions, providing better guidance in the short, medium, and long term.

## Background

In Portugal, as in other European countries, palliative care is recognized as a right, supported by Law No. 52/2012, which established the creation of the National Network for Palliative Care (NNCP) to provide active and comprehensive care to patients with serious illnesses in distress and their families. The NNCP is coordinated by the National Commission for Palliative Care, in conjunction with the Regional Health Administrations, and includes different types of units/teams for specialized palliative care, whether in hospitals, the community, or at home [1–4].

Despite differences in the planning and implementation of these care services across various health systems, particularly in developed countries known for providing advanced and integrated palliative care within their healthcare networks, there are common measures to prioritize home-based care. These measures include financial incentives and local arrangements to address palliative needs [1–4].

Research findings support the context of primary health care as an environment for promoting actions that enhance the quality of life for individuals with chronic illnesses and their families. Early integration of both generalist and specialized palliative care at this point in the healthcare network contributes to symptom control and care coordination. Providing continuity of care at home is cost-effective for healthcare systems, resulting in reduced emergency room visits and hospitalizations, while also mitigating disparities in access to palliative care [5, 6].

However, promoting palliative care at home requires certain conditions to be met. It begins with having a capable family member who can provide assistance to the patient in the absence of social and health care professionals responsible for home-based care. Consequently, implementing home-based palliative care programs can be challenging, especially in situations where individuals who live alone or older individuals are accompanied by other older individuals [7, 8].

In addition to the challenges related to families’ social context, other weaknesses within the healthcare system itself, such as resource scarcity and deficiencies in professional education, particularly in the realm of generalist palliative care, hinder the transformation of practice, even with established legal rights. Although supported by formal policies, achieving excellent palliative care across all points of the healthcare network, with a special focus on home-based care, remains a global challenge [2].

Models of integrated care, backed by positive experiences, involve primary health care, specialized home care, and hospital-based services [6], in addition to social care. These models, which connect all nodes of the healthcare network and rely on the presence of a capable family member, facilitate meeting patients’ preferences and needs, whether for dying at home or in healthcare institutions. Furthermore, those who receive support from formal caregivers are more likely to realize their preference for dying at home, especially when they are assisted within integrated care models that enhance primary healthcare services [7, 9].

Despite these advantages, professional education deficiencies pose challenges. The lack of skills and knowledge regarding generalist palliative care among family physicians and other health care professionals compromises the implementation of palliative care at home. Additionally, the reluctance of some specialists, such as oncologists, to refer patients early for specialized palliative care further complicates the process. These challenges are even more pronounced when there is a breakdown of care coordination among health care providers, confusion regarding the roles and responsibilities of each professional within the network, and a lack of consistent monitoring and evaluation of the implementation of strategic plans for developing palliative care [10].

Given these immense challenges already documented in the literature, studies that provide valuable data on the experiences in implementing public policies for palliative care, such as in the case of Portugal, are important. These studies continue to contribute to the development of programs and the improvement of clinical practices. Therefore, we aim to analyze the implementation of palliative care in primary health care in Portugal.

## Methods

### Study design

This was a qualitative, descriptive and exploratory study [11]. In structuring the article, we followed the *Consolidated Criteria for Reporting Qualitative Research* (COREQ) [12].

### Setting

A study was carried out in Portugal, a country with an advanced level of development in the integration of palliative care within the National Health System (NHS) [2, 13]. The project is registered at the Lisbon Centre for Research, Innovation and Development in Nursing (CIDNUR), of the Lisbon Nursing School (ESEL).

### Participant recruitment

The inclusion criteria were as follows: being an active health care professional for a minimum of six months in the field of palliative care, encompassing clinical practice, teaching, or research, in the Lisbon/Tagus Valley region. No exclusion criteria were applied. Professionals on vacation or on any form of leave during the data collection period were unable to participate.

Participant selection followed the snowball sampling technique. As the ESEL was the study’s headquarters, the first participant was a faculty member who initiated the process of recommending new participants. Each participant was then asked for three additional recommendations to expand the study’s sample.

In this progression, beyond the ESEL setting, participants were recruited from other universities and services, including the teams that make up the NNCP, such as the Community Support Teams in Palliative Care (CSTPC), and Intra-Hospital Support Teams in Palliative Care (IHSTCP). Additionally, professionals from Family Health Units (FHU), Integrated Continuing Care Teams (ICCT), and nursing students enrolled in the first professional master’s program focused on providing care to individuals in palliative situations at ESEL also participated. These students were included to broaden the approach from an educational perspective, integrating theory and practice, as well as diversifying experiences.

### Data collection

Two data collection techniques were employed sequentially: initially, semistructured interviews were conducted (Table 1), followed by focus groups. For the focus groups, participants were presented with a process flowchart for home-based care of patients with palliative needs, developed based on interviews testimonies and the Portuguese Palliative Care Law (No. 52/12). Subsequently, the following question was posed: How can palliative care be strengthened within primary health care in Portugal? In response to this question, the group was prompted to identify strengths, weaknesses, opportunities, and threats, using the SWOT matrix.

**Table 1:** Semistructured interview questions

In	Interview questions
1	Discuss the development of palliative care in primary health care.
2	What factors do you believe could make this work easier or facilitate it?
3	What challenges might arise or hinder the implementation of palliative care?
4	Comment on the political and legal aspects related to the right to palliative care in Portugal.
5	How can the experience of organizing and providing palliative care in primary healthcare services in Portugal benefit the international community?

In both the individual interviews and focus groups, we applied an instrument to characterize the sociodemographic and professional profiles of the participants. Throughout the data collection process, circular questions were used whenever necessary to further explore specific issues, as documented in the field notes.

Individual interviews took place in reserved rooms, either in the offices of the respective interviewed faculty members, in meeting rooms in the professionals’ workplaces, or in external environments according to the participants’ preferences. In all cases, the environment was quiet and private. One of the focus groups was conducted in a classroom at ESEL, while the other took place in a meeting room at FHU.

All data collection, interviews and group moderation were conducted by the first author, a nurse and nursing faculty member at a Brazilian public university, who was pursuing postdoctoral research at ESEL.

The first participant to be interviewed was recommended by the study supervisor and was initially contacted via email, along with other potential participants. Subsequent conversations occurred through instant messaging applications on mobile phones. The invitation was always preceded by an explanation of why they received the contact, an indication of who referred them, and a brief introduction to the lead researcher (affiliation, research focus, and activity at ESEL), as well as an overview of the research and its objectives. If they agreed to participate in the study, the best location, date, and time for the interview were scheduled.

To conduct the focus group with the master’s students, a prior relationship was established by giving two guest lectures on topics included in the course curriculum, which were not directly related to this research. However, at the end of one of the lectures, the research objectives were presented to the class, and an invitation to participate in the focus group was extended.

For the focus group with a CSTPC, the initial relationship was established with the manager, who, after being interviewed, volunteered to assist in inviting potential participants and scheduling the focus group at a time and place where everyone who agreed could gather at the FUH headquarters. In the same manner as the previous group, the research objectives were presented to the professionals, thereby marking the commencement of the scheduled activity. All data collection, whether individual or group, was performed in person.

The individual interviews were conducted first. Twenty professionals received the invitation, with a reminder sent ten days after the initial contact in case of no response. In total, ten professionals were interviewed. Seven did not respond to the contacts, and three had an initial conversation, but were unable to continue due to scheduling difficulties. The data collection period spanned from March to October 2023.

The interruption of the interviews was discussed and guided by the achievement of a saturation level based on the inductive thematic saturation modal, which is based on the emergence of new words [14]. Only after the completion of the interviews, were the two focus groups organized, one with six master’s students, two of whom also participated in the interviews, and the other with CSTPC professionals, two of whom also participated in the interviews. In total, 18 professionals participated.

The audio recordings of the interviews and focus groups were transcribed in their entirety and presented to the participants for validation. Changes included providing the full description of acronyms and clarifying some terms from Portuguese (Portugal). The first group lasted 75 min, and the second lasted 57 min. The average duration of the interviews was 42 min.

### Data analysis

The sociodemographic and professional profile data were encoded to generate the identification line that separated each interview and focus group from the global corpus analyzed by Alceste^®^ software (2012 version), with parameters in the standard configuration.

Alceste applied lexicographic analysis, based on the frequency of occurrence among terms and their co-occurrence. Each of the ten interviews was considered an initial context unit (ICU), as were each of the two focus groups. The corpus was organized into a single Microsoft Word^®^ file and saved on the first author’s drive, protected by a personal password.

The analysis by Alceste resulted in different lexicographic classes, each presenting a joint occurrence frequency of themes, measured by the chi-square value (Phi), and based on specific algorithms for the structural organization of the text.

This grouping by semantic roots of words in each class was represented by the fragmentation of the text with the parts of the speeches representative of each class, differentiated by colors, and named elementary context units (ECUs). The crossing of the lexicons of each ECU guided the definition of the classes.

The data interpretation was carried out based on correspondence factor analysis (CFA), which comprises a statistical analysis applied by Alceste to demonstrate the patterns of association between terms and classes. In addition to CFA, data interpretation was based on descending hierarchical classification (DHC), which consists of the algorithm responsible for hierarchically organizing the terms into classes.

### Ethical considerations

Ethical aspects were respected according to Resolution N^o^. 466/12 of the Brazilian National Health Council, and the project was approved in December 2022 with Opinion No. 5,834,865, which was accepted by the Ethics Committee of ESEL as sufficient for the development of the study in Portugal. All participants signed the informed consent form. All the instruments applied in the study were adapted to Portuguese from Portugal and reviewed by the postdoctoral supervisor, for which we did not perform a pilot test. Participants were coded as Ind (Individual), followed by the interview order number, for anonymity.

## Findings

Of the ten professionals interviewed, three were female physicians: one was a family physician from FUH, another was a physician from the IHSTCP, and the third was a physician from the CSTPC. The remaining seven professionals were nurses: one was a dual-role nurse at the CSTPC and ICCT, one was a nurse from the IHSTCP, one was from the CSTPC, and four were educators. Only one nurse was male. The average age was 45 years, with the youngest being 33 years and the oldest 57 years.

Regarding their experience in palliative care, five had been practicing for more than ten years, three for six to ten years, and two for one to five years. For their highest academic qualifications related to palliative care, four held doctorate degrees, three held master’s degree holders, two were currently pursuing master’s degrees, and one was a specialist.

In the focus groups, six nursing students pursuing their master’s degrees had already practiced palliative care in the context of the NNPC. They had between three and 13 years of prior experience in palliative care. The second group consisted of six professionals from the same CSTPC, including a physician, two nurses, a psychologist, a nutritionist, and a social worker.

The analysis generated four classes, from 840 ECUs, with a utilization rate of 76% out of the 1103 classified ECUs. The two DHCs maintained the same subdivision. The first block corresponds to class 1, which has a contextual and conditional character. The second block is characterized as managerial-assistive and is further subdivided into two subblocks. Within this, class 2 includes specificities related to home care. Both classes 1 and 2 align with the objective of this study. Class 1 comprises 42% of the corpus, with 349 ECUs, while class 2 constitutes 35% of the corpus, with 298 ECUs.

Class 1 involves the contextual and conditioning factors for palliative care in primary care, with emphasis on community participation, availability of human and material resources, health literacy, professional education, recognition of palliative needs and their coverage based on network care. The main lexicons for the DHC of class 1 are presented in Table 2.

**Table 2:** Main lexicons of the descending hierarchical classification of class 1

Class 1					
Lexical	Phi	Number of occurrences in the class	Number of ECU containing the word	Total of ECU classified containing the word	Percentage of ECU containing the word
Law/Laws	0,19	26	22	22	100%
Healthy/Health	0,18	76	61	89	69%
Population(s)	0,18	23	22	23	96%
Area(s)	0,18	42	37	47	79%
Right(s)	0,18	44	27	31	87%
Form/Formation/Formal/ Form	0,18	106	75	118	64%
Human(s)	0,16	18	16	16	100%
Think	0,16	32	28	35	80%
Plan/Planing	0,15	34	27	34	79%
Research	0,15	19	15	15	100%
Strategy	0,15	16	16	17	94%
Practice/Practically	0,14	13	13	13	100%
Organization/Organize	0,14	24	21	26	81%
Respond	0,14	15	14	15	93%
International	0,13	11	11	11	100%
Level	0,13	21	18	22	82%
System(s)	0,13	27	24	32	75%
Create	0,12	12	12	13	92%
Region(s)	0,12	13	12	13	92%
Society	0,12	12	11	12	92%
Very low/low	0,11	9	8	8	100%
Integrate/integrated/integral	0,11	14	12	14	86%
Resource(s)	0,10	40	35	57	61%

The weaknesses of the NNPC in class 1 have become evident in the face of issues such as unequal coverage of palliative care across all regions of the country. The lexicons ‘law’, ‘population’, ‘area’ and ‘right’ underscore this situation.

*“Our problem does not lie in legislation; it lies in operationalization. We are a country that recognizes the right of others to benefit from this type of care*,* given the vulnerability associated with advanced illness and end of life conditions. However*,* we lack resources. The national response we have is uneven.”* (ECU No. 122, Ind 1).

Related to this problem, the capacity of the NHS to respond to the demands of an assistive network such as the NNPC, which requires investments, has come into play. The lexicons ‘formation’, ‘human’, ‘thinking’, and ‘planning’ address these deficiencies.

*“We are dealing with outdated data*,* and since plans are biennial*,* the 2021–2022 plan was delayed*,* and objectives were not met.* […] *Creating a plan within two years is challenging*,* and even if it were structured with time*,* some objectives are ambitious. It is not advisable for palliative care teams to emerge if they are not effectively constituted*,* as this would distort the concept of palliative care”.* (ECU No 435, Ind 4)

The lexicons ‘society’ and ‘very low’ highlight the global problem of insufficient knowledge about palliative care.

*“*[…] *We have a society with very low health literacy. If you ask people whether they are aware that this is a right and that they should claim it*,* most are unaware. When people do not assert this right*,* it is also not a requirement for it to occur.”* (ECU No. 122, Ind 1).

The low literacy regarding palliative care threatens the development of the field, which involves the lexicons ‘society’ and ‘very low’, associated with the need for integration.

*“To address this*,* it must be done in an integrated and coordinated manner*,* involving compassionate communities* […]. *There is no sustainable solution from an efficiency perspective that can achieve the desired results without being grounded in the health*,* social and societal axes. Everything integrates into a clear definition of a health policy.”* (ECU No. 200, Ind 2).

Among the positive points, participants pointed out the legal framework; only one participant raised the issue of the basic law, which was highlighted in the Alceste report, associated with the lexicons ‘strategy’, ‘practice’, ‘organization’, ‘system’ and ‘create’.

*“Today*,* I say that it was a mistake to create a specific basic law for palliative care. I understand that it may be important for marketing*,* but from an operational point of view*,* it creates problems. There are two laws*,* one for palliative care and one for the foundations of health; in terms of gradation and hierarchy of law*,* they are the same. Palliative care should be integrated into the basic health law because it is a response; there is no basic law of cardiology*,* mental health*,* as fragmenting what should not be fragmented”.* (ECU No. 282, Ind 2)

The lexicons ‘research’, ‘data’, ‘models’ and ‘development’, present the participants’ perspectives on Portugal’s role in generating evidence for palliative care.

*“We do not have experience collaborating with other countries in Portugal. Our health system is somewhat different at the European level. The country we are similar to is Spain; in fact*,* our model of palliative care closely resembles the Spanish one because that was where we were drawn inspiration.* […] *At this moment*,* we have teams that recognize research as one of their components* […]. *In addition*,* this evolutionary perspective is favorable.”* (ECU No. 140, Ind 1).

From the practical experience of palliative care in Portugal, the lexicons ‘resources’ and ‘training’ point to characteristics of an ideal model.

*“Two things are crucial for facilitation: one is exclusive dedication to palliative care*,* which is a problem in Portugal* […]. *In the models I am familiar with*,* there is an exclusive dedication*,* which allows professionals to focus*,* ensures prompt response times*,* and contributes to higher proficiency. The other issue is the quantity of human resources* […]. *This problem cuts across all aspects of health care; currently*,* there are no professionals available*,* so it implies that decision-makers need to reorganize the system because the majority of deaths occur due to chronic disease*, […] *to avoid overburdening hospital services* […]”. (ECU No. 174, Ind 2)

Class 2 involves patient care at home during the end of life, and the dynamics of home care favor this. The main lexicons for the DHC of class 2 are presented in Table 3.

**Table 3:** Main lexicons of the descending hierarchical classification of class 2

Class 2					
Lexical	Phi	Number of occurrences in the class	Number of ECU containing the word	Total of ECU classified containing the word	Percentage of ECU containing the word
House(s)	0,35	98	83	103	81%
Family(ies)	0,31	96	79	104	76%
Patient(s)	0,30	266	174	324	54%
Caregiver(s)	0,25	43	33	35	94%
Control/Controlled	0,22	29	26	28	93%
Passed away	0,20	22	20	21	95%
Die(s)	0,18	23	22	26	85%
Symptom(s)	0,17	23	22	27	81%
Urgency(ies)	0,16	32	24	32	75%
Query	0,15	31	21	28	75%
Know	0,14	26	20	27	74%
Able	0,14	14	13	15	87%
Situation(s)	0,14	47	44	77	57%
Telephone	0,14	23	18	24	75%
Symptomatic	0,14	13	13	15	87%
We Tell/Tell/Contact/Glad	0,12	13	11	13	85%
Desire/Desirable	0,12	13	11	13	85%
Hinders	0,12	12	11	13	85%
Depends/Dependence/ Dependent(s)/Depend	0,12	15	14	19	74%
Crisis	0,11	8	6	6	100%
Trained(s)	0,11	8	6	6	100%
Alone	0,10	8	8	10	80%
Hospital	0,10	33	33	62	53%

One of the challenges of CSTPC is to increase the time spent at home and reduce visits to the emergency room. The lexicons ‘home’, ‘family’, ‘patient’, ‘caregiver’, ‘control’, ‘symptom’ and ‘urgency’ present events that are difficult to manage, particularly in the context of oncological disease.

*“Insecure events*,* such as seizures and hemorrhages*,* lead to urgent medical attention. This is because families struggle to manage these symptoms* […]. *Regular visits to the emergency room serve as an indicator that the family is faltering*,* unable to keep the patient at home*,* and prompts the team to question whether the patient should indeed remain at home*.” (ECU No. 100, Ind 1).

The lexicons ‘consult’ and ‘know’ relate to the timing of case management, especially by specialized teams.

*“The organization of home care depends on when the patient reaches the specialized team. The later the arrival*,* the greater the likelihood of spending less time at home and resorting to urgent care. This is because the team is not yet familiar with the patient or caregiver*,* and vice versa. Trust has not been established*,* and naturally*,* insecure individuals will seek what they know best*,* which often leads them to the hospital*.” (ECU No. 97, Ind 1).

Maintaining the patient at home depends on family arrangements and professional care within the community, including the level of training of the teams and their availability to attend crises. The lexicons ‘family’, ‘able’, ‘situation’, and ‘phone’ support this information.

*“We manage end of life care at home through prevention and anticipation of situations. For some patients*,* honestly*,* we keep the phone line open for longer. The team operates from 8:00 AM to 8:00 PM.*,* and if I have a patient who appears unstable*,* I do not disconnect the phone at 8:00 PM*” (ECU No. 892, Ind 10).

The family member/caregiver needs to be available and willing to support the patient’s stay at home. The lexicons ‘desire’, ‘complicate’, and ‘depend’ underscore this issue.

*“We have people who want to be at home but lack the necessary conditions - they do not have a caregiver or sufficient resources. Additionally*,* there is a population that believes health problems are solved by others*,* institutions*,* spaces*,* or professionals*,* without recognizing the complementary nature of these factors.”* (ECU No. 390 Ind 4).

The hospital appears in the lexicon when the topic of death is addressed, and it is connected with other terms such as dependency and crisis. This is because it relates to the aforementioned aspects, including families’ capacity, individuals’ desire, the availability of specialized professional care at home, and the pathways/services offered by the network.

*“Some patients want to spend their final days at home but prefer to go to the hospital only for their passing.* […] *There are families who do not feel prepared for the patient to live at home until the end. They are isolated* […], *either because their neighbors are older and unable to assist or because they are foreigners*,* and because neighborhood solidarity is diminishing*,* affecting the ability to care until the end.”* (ECU No. 15, Ind 1).

The transition from the hospital to the home, based on the IHSTCP, involves challenges represented by the lexicons of ‘dying’, ‘crisis’, and ‘dependence’.

“*Patients often face economic hardships. If the community team does not provide an immediate response*,* it is difficult for them to return home. In such cases*,* we usually consider referring them to a palliative care unit”* (inpatient unit). (ECU No. 820, Ind 9)

When addressing pathways within a network, connections with hospitals are crucial. Social factors not only influence the management of symptoms and crises but also impact hospital utilization.

“[…] *We witness severe acute symptomatic disruptions and admit individuals with social issues to resolve*,* often leading to caregiver exhaustion. The challenge of hospitalization poses a threat to network integration*,* potentially resulting in prolonged suffering and patients ending up in emergency situations.”* (ECU No. 1015, Ind 11).

One of the strategies employed by CSTPCs has been health education, aimed at disseminating the concept of palliative care and facilitating the management of complex cases.

*“There is a team of nurses working on the training informal caregivers*,* conducting group sessions* […]. *It is an opportunity to empower the caregiver*,* allowing them to extend the time a patient spends at home*,* even if they don’t pass away there.”* (ECU No. 1022, Ind 11).

Education is thus positioned as the central core for developing palliative care in primary care, both to expand the capacity of health care professionals for generalist palliative care and to engage the population in meeting a need that affects everyone.

To increase the patient’s time at home, reduce visits to emergency rooms, and optimize the use of network resources, active family participation, along with integration with a specialized palliative care team, is necessary. In Portugal, this team is responsible for fully managing the most complex cases in home care, providing consultation to primary care teams, and disseminating technical and popular knowledge about palliative care.

## Discussion

The investment by healthcare systems in providing palliative care is a response to the growing needs of an aging population, the high incidence and prevalence of chronic diseases, and the suffering caused by these events [10]. Respecting the principle of patient autonomy in palliative care involves ensuring safe access to healthcare services wherever the patient wishes to be, and being at home is a common choice at the end of life [10, 15, 16].

Therefore, equitable access to palliative care services, distributed geographically to reduce avoidable and unjust differences, serves as an indicator of quality [10]. When addressing issues related to low literacy, deficiencies in health care professionals’ education, and resource scarcity, it is essential to consider not only territorial distribution and team capacity but also financial and cultural accessibility within rural and remote areas. Discrimination should be avoided, and culturally appropriate care should consider the needs and beliefs of diverse populations [17–19].

To expand access to palliative care, prevent hospital overload, and address the challenge of human resource deficits, healthcare systems such as Portugal’s prioritize community-based and home-based care. This approach involves collaboration between primary health care professionals and specialized services, including home care assistance [20, 21].

Operationalizing home care through the integration of generalist and specialized palliative care has yielded positive outcomes, such as longer patient stays at home [21, 22]. However, overcoming social and structural barriers to providing home care for all who need it requires strong social support [10, 23, 24] to assist caregivers and prevent burnout [25]. These dependencies contribute to the challenges faced in accessing palliative care at the primary care level.

Just as low health literacy can interfere with the development of palliative care, because people who are unfamiliar with it fail to seek it out, more literate populations on the subject strengthen bonds and structural mechanisms for internal support in compassionate communities [26], contributing to home-based care.

An observational study on the integration between primary health care and home-based palliative care units in Italy revealed that death at home is favored by the presence of specialized professionals. Even in cases where family physicians provide care, as long as they consult these specialists, patients are more likely to pass away at home. Among the patients who died during the study (*n* = 517), 184 (75.4%) of those under specialized palliative care teams died at home, while 180 (65.7%) of those attended by family physicians also passed away at home [7].

To align with a policy supporting death at home, planning with short-, medium-, and long-term goals, including professional training and health education, is necessary. Our findings demonstrate how a poorly timed strategic plan for operationalizing objectives, such as education, can impact provider trust and user satisfaction. Patient and family dissatisfaction poses a threat, as experiences falling short of expectations can interfere with the very process of literacy and acceptance of palliative care.

Our data criticize Portugal’s biannual strategic plans, arguing that a two-year period for a plan aimed at developing palliative care in the country is insufficient to meet its needs, and thus it has not achieved all its goals. Strategic plans by major health organizations, such as the World Health Organization, seek to ensure a long-term vision while maintaining the capacity for ongoing revision and adaptation to changing needs and environments.

Our data reinforce how developing palliative care is a process that requires time and effort. While there is not a rigid and rapid deadline, as it can vary depending on available resources, it is essential to allocate time for a comprehensive, sustainable, and inclusive approach, which necessitates ongoing situational analysis.

However, another noteworthy finding-one of the motivators for this study-is the lack of publicly available data that would enable measurement and evaluation of the achievement of goals and objectives in these plans. Making decisions to improve the quality of care and even adapt plans requires an understanding of operations and their interplay. For a systemic assessment of access to palliative care, it is crucial to comprehend how these indicators relate to each other [27]. Measurable indicators include the number of patients who die at home and the number of home visits by the specialized team, which are associated with the continuity of home-based care and the capacity of the home care program. A positive correlation between these indicators suggests that the program is fulfilling its mission [5].

In terms of continuity of care, our data confirm that the community team’s response needs to be swift, especially when dealing with oncology patients; otherwise, it is unlikely that the patient will be able to return home. This aspect, influenced by service availability and responsiveness, directly impacts patients’ real choices regarding their place of death. According to other research findings, the later a patient is referred to palliative care, the worse the quality indicators tend to be [5, 28, 29].

In general, the critique of strategic plans raised by participants, coupled with the lack of publicly available data from the NNPC, reflects how being well-ranked in the Global Atlas of Palliative Care does not guarantee equity and quality in palliative care [2]. This classification does not fully assess reality in an organic and comprehensive manner; it is an important step for recognizing each country’s investments but still faces many operational challenges.

This situation appears to be common to the reality of other countries. In the United States, for instance, which is also classified as level 4b for advanced and integrated palliative care, palliative care is utilized for 45% of all deaths, with a median of only 17 days before death [4], which is essentially linked to its application at the end of life.

When addressing health rights, particularly the right to receive palliative care as early as possible [7], tailored to the evolving palliative needs from the diagnosis of the chronic disease-causing suffering, our data highlight the need for specific legislation. Although its importance for visibility in the field is acknowledged, it also introduces a perspective of overlapping rights and conflicts that may impact resource distribution and effective implementation.

We understand that a specific foundational law for palliative care should not be based solely on being an isolated specialty. The approach to palliative care differs from other medical specialties because it aims to care for the whole person, alleviating suffering and supporting quality of life, regardless of diagnosis and patient age [19].

It is crucial that this approach be adequately regulated, organized, and funded to ensure that its core aspects, such as professional training, human resources, exclusive dedication, and 24/7 availability, among others, are addressed. A formal policy for palliative care does not diminish the importance of other specialties; rather, it helps establish the necessary legal framework for collaboration among experts. Patients in palliative care often have complex needs and may traverse the entire healthcare network.

Limitations include the data being collected in a single region of Portugal and participant recruitment through snowball sampling, which may introduce selection bias by resulting in a sample susceptible to the characteristics of a specific group, limiting the perception of different perspectives. Furthermore, most participants were nurses, although these professionals are the most numerous in the teams and dedicate significant time to direct assistance to patients and their families in al contexts, such as in primary health care.

## Conclusion

Our data highlight the importance of evaluating and monitoring interventions for palliative care within the health care network, particularly in primary care settings where access inequities are observed. Measurable indicators guide goals and resource distribution, and the presence of specific legislation ensures the promotion of good practices across all medical specialties, recognizing that everyone experiences illness and death. Despite some weaknesses and external threats, along with advanced legislation, our analysis concluded that Portugal serves as an example in this field due to its comprehensive approaches and commitment to prioritizing palliative care for investment, professional training, and health education.

## Data Availability

The original contributions presented in this study are included in the article materials, further inquiries can be directed to the corresponding author.
